# A Narrative of a Journey to London in 1814, or a Parallel of the (between) English and French Surgery, &c.

**Published:** 1816-10

**Authors:** 


*Relation d'un Voyage fait a Londres en* 1814*; ou Parallele de la Chirurgie Angloise avec la Chirurgie Françoise, &c.* Par PHILIBERT JOSEPH ROUX, &c. &c.

---

*A Narrative of a Journey to London in* 1814, *or a Parallel of the (between) English and French Surgery, &c.* By P. J. ROUX, &c. &c. Translated from the French.

THE *Second Part* of M. Roux's Narrative now remains for examination. It is entitled *Surgical Doctrine and Practice of the English.* Upon the plan instituted in the former part of our Review, we shall concentrate within distinct sections whatever of the novel or instructive it may afford. To those passages in which the defects of English Surgery are the subject of sound and enlightened criticism, we shall advert with peculiar care; and pause on every point which most strikingly exhibits the characteristic differences of our own nation and the French in the theory and practice of the science.

*Dressing and mode of operating of the English Surgeons.* M. Roux describes our *mode of dressing wounds and ulcers* as very simple. English surgery, indeed, is, like the French, and in a much greater degree than our medicine, destitute of the poly-pharmaceutic character. This arises not only from our rejection of unguents and compound topical applications, but from our singular predilection for immediate union of even the most extensive wounds. Our treatment of sores by the adhesive strap tends farther to promote this simplicity. We are absolutely in want of many of the requisites for surgical dressings, or they are difficultly procurable. French *charpie* is greatly superior to our lint for defending, and absorbing the moisture of, recent or suppurating wounds; and, in filling up the hollows and inequalities of a sore, this *charpie* is employed by them instead of tow. Compresses are little used by us; and bandages only with a view of exercising compression. The adhesive plaster, whenever its action will suffice, is our sole retentive. Needless pains are taken to remove the products of suppuration by frequent sponging and ablution of sores. The nature of the dressings applied by the French, their care to institute expulsive compression and counter openings in deep wounds and abscesses renders this practice still further unnecessary. They clear the surface of sores with *charpie,* and the circumference with linen. In the *general mode of operating* of our surgeons, M. Roux finds much to censure. This term he restricts not to the mere performance of an operation: it includes the conduct of the surgeon in every thing which immediately precedes and follows the act, and even the principle which directs it. The charge preferred against us, of deciding rashly on operations is, he allows, unfounded. We are more bold, enterprizing, and fond of new and extraordinary experiments than the French; but few of our discoveries and inventions have been sanctioned by experience. They, on the other hand, know how to preserve themselves from enthusiasm in the culture of the sciences, and to be on their guard against "extravagant inventions, false systems, and erroneous doctrines." Our patients are seldom submitted to any preparatory plan of food or medicine when a surgical operation is to be performed—a precaution invariably observed by the French in cases admitting of the necessary delay; nor do we allow them time, as is the custom of the latter, to become previously inured to the air of the hospital or to the moral influence which its scenes are calculated to exert. With regard to the operation itself, we are not sufficiently careful in excluding from the patient's view the apparatus of surgical instruments, and obviating whatever may prove the source of mental inquietude and agitation. Our surgeons, unlike the French, commit to assistants the application of the tourniquet and dressing of the consequent wound. They also evince an excess of coldness and indifference towards the patient, and consume in the operation much more time than is absolutely requisite for its completion. Such, at least, are the results of M. Roux's observation; and some of them, it must be acknowledged, are but too correctly drawn.

*Wounds* or "recent solutions of continuity" are remarkable as succeeding alike surgical operations and fortuitous injuries. The surgeon indeed, in his operative processes, imitates almost every species of accidental wound. Not only do his knife and saw implicate the various kinds of structure which enter into the composition of the external organs, but even the viscera of the great cavities are not beyond the reach of his adventurous hand. The only difference between accidental wounds and those consequent on a surgical manœuvre, is in the mode of infliction. In nature, indications of cure, and treatment, they are the same; except that the wound of an operation, being made on fixed principles, exhibits less variety of form and character than the adventitious, and affords data, more positive and precise, for the institution of a remedial plan. And we may add that it is never, or only in a trifling degree, complicated with laceration or contusion. The prejudice of our surgeons in favour of union of wounds by the first intention is extreme: they employ it with indiscrimination, blind as extravagant, in all cases; while the French retrict its application with a "perhaps too rigid economy." But we, poor souls, are driven to this pernicious resource by want of those requisites for surgical dressings which France possesses in profusion: we are simple and frugal from necessity! Thus one of the most splendid achievements which the history of Bristish surgery can exhibit, sprang from the dearth of linen rag! What solemn trifling; what low and despicable sophistry is this! But to resume the thread of analysis.—Immediate re-union of wounds is, like all other good things, subject to abuse, and not susceptible of application to every case in which it may be accomplished. It is contra-indicated, for example, in the operation for removal of a testicle; because high inflammatory action is sometimes induced by irritation of the necessary sutures, and the hæmorrhage, occasionally taking place from small vessels to which no ligature can be applied, and consequent infiltration of the cellular texture of the scrotum with blood, can only be obviated by plugging the interior of the wound, a plan obviously incompatible with views of direct re-union; and, moreover, the completion of the healing process is not really accelerated by an attempt to procure re-union in such cases. How far this latter assertion is correct, experience, such as few British surgeons possess, could alone enable us to decide. Again, immediate re-union should not be attempted in the wound made in the operation of aneurism: and wherefore? Because, forsooth, it unfortunately precludes, or at least does not exactly favour, the employment of those precious things called ligatures of reserve! This is the blind leading the blind; this is the owl criticizing the vision of the eagle, with a vengeance! We did hope that M. Roux would have carried back with him from the London schools, some of our correct notions on the operation and employment of the ligature in aneurism, and taught his countrymen to abjure all those pernicious contrivances against secondary hæmorrhage, which serve only to induce the mischief they are intended to obviate. Lastly, immediate re-union of the wound after amputation of a limb is not, in all cases, to be attempted. But what are the peculiar circumstances contra-indicating it, M. Roux does not deign to inform us. The plan of cutting off both threads of a ligature close to the knot, in order to secure perfect re-union of a wound, as proposed by Delpech,* and subsequently practised in this country by Lawrence,† is mentioned in terms of commendation. Roux has thrice tried it after the amputation of cancerous mammæ; and in none of the cases, observed any unpleasant consequences to result. Whenever it is adopted, great precaution should be used to tie each vessel sepa-

* *Memoire sur la pourriture d'hôpital.* Paris, 1815.

† *Medico-Chirurgical Transactions*, vol. vi; *This Journal*, vol. ii, page 28. Rev.

rately, and the ligature applied very near its mouth; so that the portion of artery left below the ligature, and hence destined to slough away, may be as minute as possible.

*Ulcers.* It is difficult to establish a correct distinction between wounds and ulcers. The former are frequently converted into the latter by the operation of some local or constitutional cause. Ulcers frequently succeed wounds. The former, however, may be distinguished by the density and livid colour of the surrounding integuments, and by their comparative tardines to cicatrize. Rest in the horizontal posture, with the application of *charpie* and large emollient cataplasms, changed once or twice a day, according to the state of suppuration, constitutes the general plan of French treatment of ulcers on the legs. Temperate regimen and internal tonics are prescribed as auxiliaries. When the curative process is so far advanced as to admit of cicatrization, the cataplasms are laid aside, and the sore dressed as a simple wound, with care to repress the surface by occasional applications of nitrate of silver. Sometimes tonic plasters are had recourse to; and their action is aided or supplanted by the employment of a "slightly compressive bandage." The English method of treatment by the adhesive strap is next minutely described: and M. Roux very candidly acknowledges the great and unexpected advantages which have resulted, in his hands, from the use of this simple remedy. He concludes by detailing several cases in which he has employed it with signal success.

*Fractures.* In our Review of Mr. Cross' "Sketches," we have traced many of the more striking peculiarities of French practice in the treatment of fractures; and we verily believe that, in this department of surgery, the palm of superiority may, with justice, be claimed by our rivals. To the genius of Desault they are deeply indebted for the precision and success, which the art, in this respect, at present boasts. The French, it will be recollected, constantly adopt, in fractures of the lower limbs, the extended and horizontal posture, except under particular circumstances which we shall hereafter specify. Not "one good surgeon" among them, according to M. Roux, is an advocate of Pott's method. The objections of the Frenchman to the half-bent position of the limb are principally the following: It is of no use in subduing the spasmodic contraction of the muscles which sometimes opposes the reduction of fracture; because the reduction may, with impunity, be deferred until such spasm has ceased spontaneously, or yielded to the resources of art: it offers no advantages in obviating consecutive displacement, which may not be more completely obtained by the exact and uniform pressure of the French apparatus; and, in oblique fractures only can such an occurrence be apprehended: it is mischievous, in one instance,* with respect to the stiffness and immobility of the joints consequent on long repose; in others, it does not prevent or palliate the inconvenience: lastly, it is particularly objectionable, as rendering difficult the investigation, occasionally requisite, with regard to the length of the limb, and direction of the bone in femoral fracture, and precluding the extension of the retentive apparatus from the thigh to the leg. In fact, the half-bent and lateral position of the limb is only correctly indicated when the osseous lesion is complicated with a wound of the soft parts; such as, from its situation, would be more readily accessible, without disturbance of the limb, in this than in the extended posture. We would beg leave to add, from personal observation, that, whenever the semi-bent and lateral position is determined on in fracture of the thigh, the patient should be laid fairly upon the side and not on the back, as is too frequently practised among us, even by judicious surgeons. The consequences of this inattention are, that precise correspondence between the extremities of the broken bone can never be maintained, and the limb invariably acquires some degree of twisting or distortion. M. Roux here renews the controversy, of late sustained by the French and English surgeons, respecting re-union of the neck of the femur in cases of fracture. The nature and merits of this question have been elsewhere so clearly defined, and ably stated by Mr. Cross,† that we deem any farther discussion of it, in this place, unnecessary; nor is any new fact or argument adduced by the Paris champion in support of his cause. The frequent occurrence of false articulation after fracture of the limbs, in this country, is regarded by M. Roux as a proof of the negligence or inferiority of our surgical treatment.

---

* In the joint *below* the limb on which the fracture has been inflicted, the stiffness is always most remarkable, and obstinate of removal. Hence, when, for fracture of the *thigh*, the limb has been placed in the *half-bent* position, the patient will be obviously rendered incapable of putting his foot to the ground so long as the false anchylosis of the *knee-joint* continues. By the extended position of the limb, this inconvenience is prevented.

† *Sketches of the Medical Schools of Paris,* page 88. *Med-Chir. Journal and Review,* vol. i, page 258. Rev.

Such accidents are rarely seen in French practice. For several years past, he asserts, the operation of removing the extremities of a bone, unconsolidated after fracture, has not been performed in France; and he has known no instance of the seton having been employed for that purpose, as recommended by Dr. Physick, and recently tried by Wardrop, Bell, and Brodie* here. Of the cases operated upon by the two latter gentlemen, a minute history is detailed by M. Roux. The superiority of the operation by seton to that effected by the saw, must be sufficiently obvious.

*Dislocations.* French surgeons may justly "claim the glory" of having, in a singular manner, extended and brought to perfection a system of doctrine of luxations, and simplified to the utmost, the process of reduction: while the English have shewn themselves more enterprising in certain complicated dislocations, particularly those of the elbow and ankle-joints. This is nearly all we can glean from M. Roux on the present subject. He passes a high and honourable eulogium on the "Observations in Surgery" of the venerable Mr. Hey, of Leeds. Truly may it be said, that his work "contains many valuable facts bearing the stamp of the soundest surgery."

*Fungus Hæmatodes.* Much difference of opinion seems to exist between English and French pathologists respecting the precise application of this term. M. Roux asserts that most of the cases pointed out to him, in London, as specimens of this affection, were merely a variety of carcinoma, the soft fungous cancer; not preceded, like the more common form, by a state of scirrhus. He denies the existence of what we call Fungus Hæmatodes, as a specific or peculiar malady.

For "certain varieties of cancer, sanguineo-fungous tumours, and white swellings of the joints, have been wrested from the different genera to which they belong, and their assimilation attempted in order to constitute a particular genus of disease: they are so many different individuals belonging to several families, of which it has been wished to compose a new family."

The term, fungus hæmatodes, is regarded by the French surgeons as synonymous with sanguineo-fungous tumour; and this latter includes not only our aneurism from anastomosis, but the varicose tumour which frequently derives its origin from congenital marks of the skin. Several

---

* *Med-Chir. Transactions,* vol. v. Rev.

cases and observations are subsequently detailed, to elucidate the structure, varieties, developement, and conduct to be pursued in the treatment of this disease. One variety of it, known by the name of Pott's Aneurism, is described as resulting from extensive dilatation, and sieve-like perforation, of the coats of one middle-sized, or several smaller arteries, and consequent injection of the blood, not into one cavity but into the interstices of the cellular, and even the muscular structure: thus the whole is converted into a spongy mass. Other tumours of this genus arise from dilatation of the capillary vessels, arterial as well as venous, which extends gradually to the larger branches from whence the capillaries proceed. Some of them, as resulting from dilatation of the veins only, are simply varicose; but commonly they possess a mixed character, and partake more of the nature of aneurism or varix, according as one set of vessels or the other is most deeply implicated. The capillary arteries are never alone affected. In some cases, particularly the simply varicose, the tumour remains stationary when it has acquired a certain volume; in others, its progress is fearfully rapid. M. Roux suggests the propriety of the early destruction of *nævi materni* by caustic, compression, or the knife, so as to obviate any future developement of a fungous tumour. Compression can only be exercised with advantage when the skin-spot is situated over a bone, and will be less certain in its results than the other means. It was, however, attended with perfect success, in the case of one of M. Roux's children, aged four years, who had a congenital mark on the right temporal region. About the second month, the system of compression was commenced by means of a pad and elastic bandage, and continued without intermission for three years. The only trace of the spot now remaining, is a "faint violet zone," which corresponds with the circumference of the original nævus; and in the centre of which the integuments have resumed their natural appearance.

*Aneurism.* Very little, calculated to interest or instruct the British surgeon, presents itself in this section. The people of England are represented as more prone to external aneurism than their continental neighbours; and the cause of it is attributed to the "nature of the labours" in which they are engaged. In tracing a rapid historical sketch of the operation for aneurism, another attempt, futile as ungenerous, is made to tarnish the reputation which our illustrious countryman, John Hunter, has acquired in this department of surgery; but the confession is reluctantly extorted from M. Roux, that we have, from the commencement of the last century, "contributed more to the progress" of it than the French. Aneurism from anastomosis is one of the terms which they apply to the affection, designated by the English aneurismal varix; and with our varicose aneurism, they seem to possess but a very limited and imperfect acquaintance. There are cases of aneurism to which they yet consider the treatment by incision of sac applicable; and M. Roux imagines that they will long retain the employment of "broad flat ligatures, and ligatures of reserve." We shall pass over the detail of several cases of aneurism operated upon by M. Roux, Travers, and Cooper: part of them present only a mortifying spectacle of French blindness and bigotry; and the others are consigned in a work with which no British *Surgeon* can, by this time, be unacquainted — the valuable Treatise of Mr. Hodgson. In our review of that publication,* we, it will be remembered, noticed a new plan, proposed by Dr. Jones, for the obliteration of arteries, and subsequently modified by Mr. Travers. We also stated, that this plan had been unsuccessfully tried on the human subject, by Dr. Hutchinson, of Deal Hospital. A rumour, at the same time, was afloat, that Mr. Astley Cooper had made the same experiment, with results equally inauspicious. Our distance from the metropolis precluded the necessary inquiries respecting its authenticity; and all recollection of the circumstance had well-nigh died away, when it was re-awakened by the distinct assertion of M. Roux, as to Mr. Cooper's failure. Mr. Travers will, we hope, *contrive* to bear in mind these cases, and, like us, consider the evidence with which they are fraught, as decisively against the repetition of a cruel, because needlessly perilous, operation.

*Diseases of the Eye.* The prejudice of the English people, in favour of oculists, is greater than that of the French. Our surgeons are little solicitous about re-attaching the surgery of the eyes to general practice. In France, the reign of the oculists is drawing to a close; and this branch of art will be once more united to the trunk from which it ought never to have been separated: for all the more important discoveries and innovations in the treatment of ophthalmic diseases we are indebted, not to the oculist,

* *New Medical and Physical Journal*, vol. 10, p. 499. Rev.

but to the general practitioner of surgery. Of the three modes by which an artificial pupil may be formed, simple incision of the iris, excision of a portion of the membrane, and detachment of a part of its circumference, none is entitled to exclusive adoption: each of them is capable of advantageous employment according to the peculiar circumstances attending the operation. M. Roux, in a fruitless attempt to depress an adherent cataract, unintentionally detached the iris in a portion of its external circumference, and thus formed an artificial pupil, which afforded passage to the light; and the patient left the hospital with his vision so far restored as to be capable of distinguishing many small objects. The pupil of the other eye was darkened by incurable albugo. The plan proposed by Sir W. Adams for the cure of ectropion, by removal of a triangular portion of the eye-lid, M. Roux evidently does not consider preferable to the more simple process of excision of the tumefied conjunctiva. To the institution of dispensaries for diseases of the eye, he raises some rather plausible objections. The multitudes of people who resort to them; their necessary exposure, often under unfavourable circumstances, to all vicissitudes of weather; the comparative shortness and too rare recurrence of the interval devoted to consultations; and the influence which such a system evidently exerts in widening the separation of ophthalmic from general surgery;—all contribute, in M. Roux's view of the subject, to render these institutions injurious rather than productive of public good, hostile to the habits of correct observation in the surgeon, and, of course, unfavourable to the progress of improvement in the science.

*Fistula in Ano.* M. Roux expresses surprize, that, in performing the operation for this disease, the English surgeons prefer the employment of Pott's bistoury to that method, which consists in passing a common straight bistoury upon a grooved director, open at the extremity and traversing the fistula, while its remote point rests in the groove of a gorget of wood introduced into the rectum. This apparatus is represented as greatly superior to our curved bistoury, and applicable, with little or no modification, to every variety of the disease. The director should be made of steel, and slightly pointed. The French, after the operation, introduce a pledget of *charpie* into the intestine, above the boundary of the incision, and insinuate it between the lips of the wound. This is renewed every day, with care to lessen its bulk in proportion as the wound fills up. The English surgeons, on the other hand, content themselves with separating the lips of the wound by a strip of linen, and soon, with the exception of cleaning it, abandon the sore to itself. The recurrence of fistula, after the operation on the French plan, is very uncommon.

*Chronic venereal Enlargement of the Testicle.* It has been remarked, by some English practitioners, that this species of swelling commonly assumes a pyramidal form. The experience of M. Roux confirms the general correctness of the observation. But this peculiarity of figure is not exclusively confined to the venereal affection. Sarcocele sometimes exhibits it; nor should its presence be regarded as decisively contra-indicating a scirrhous or cancerous state of the organ.

*Cancer of the Penis.* Three cases only of this kind have fallen under the eye of M. Roux for many years past; and in all of them he remarked the existence of natural phymosis, for the removal of which not one of the patients had submitted to the usual operation. Hence he has been led to infer, that phymosis may generate predisposition to cancer of the glans. The observations of Mr. Hey serve singularly to strengthen this inference. Out of twelve cases, in which amputation has been performed by him for cancerous penis, nine are expressly said to have been preceded by phymosis, natural or acquired; which subsisted till the developement of the malignant malady.* The practical conclusion is, that on the minds of all persons troubled with phymosis, the necessity of submitting to an operation for its removal should be strongly impressed. Review of all these cases seems to countenance an opinion, that cancer of the penis is an affection purely local, and hence less frequently reproduced than cancer of other parts.

*Diseases of the urinary Passages.* The steel sound is employed by us to ascertain the presence of stone in the bladder. On one account, the silver catheter, which we exclusively reserve for the evacuation of urine, is preferred to the sound by M. Roux: it will enable the surgeon, after he has explored the full bladder, to relieve the viscus of its contents without removing the instrument. Yet the Frenchman candidly acknowledges that the sound, from

* *Practical Observations in Surgery*, p. 461, Rev.

its greater weight and solidity, may be more advantageously used in detecting the existence of a small calculus; and that, with it, will be avoided the delusive sensation communicated to the operator's finger by the rushing of the urine into the catheter, on its entrance into the bladder, or the agitation of the fluid in the canal of the instrument.—*The prostate gland,** although prone to various forms of morbid affection, particularly inflammation terminating in suppuration and chronic enlargement, is not so frequently the seat of disease, and hence productive of retention of urine, as English surgeons imagine. In the treatment of *urethral stricture and its consequences,* our surgeons are greatly behind the French. In proof of this, we are represented as frequently practising incision of the urethra in the perinæum (l'operation de la boutonniere), to obviate retention of urine when any obstacle to the introduction of the catheter exists, and even where the case is not complicated with urinary abscess or infiltration. An expert surgeon, if the patient possess requisite courage, will never, or rarely, fail to overcome the obstruction, especially with the conical catheter: and hence the operations of opening the urethra in the perinæum, or puncturing the bladder for retention of urine, is seldom found necessary by able practitioners in France. The treatment of stricture by the caustic bougie is rapidly declining in England.

*Operation of Lithotomy.* Nothing advanced on this subject by M. Roux, merits particular attention. In English as in French surgery, this operation is said to have attained the highest perfection of which it is susceptible. We should be glad to support this opinion with our own testimony; but can only do so when the "operation of Cheselden, in all its purity," shall be universally practised by the surgeons of "continent and isle," to the exclusion of the *lithotome caché de frère Côme,* and of the blundering and unscientific gorget.

*Hernia.* After some frivolous observations on the institution, formed in London, for supplying the ruptured poor with trusses, M. Roux enters into a concise and tolerably

---

* "That glandular body, which surrounds the neck of the bladder and commencement of the urethra in man, and which we name the prostate." This is a literal translation from the *original,* page 312. We select it as a fair specimen of the diluted style of composition which the French writer adopts. Rev.

clear description of "the anatomy of the crural arch and inguinal region;" but this is ground with which the splendid work of Mr. Cooper, and "didactic treatise" of Mr. Lawrence, have rendered British surgeons so familiar, that we need not retrace it. The latter production is mentioned by the Frenchman in terms of high commendation.

*Amputation.* We did hope and expect to have found here correctly defined and specified, the peculiar circumstances which contra-indicate, according to French notions, immediate re-union in large wounds; and this anticipation was not a little heightened by the profession of a design to "collect under one point of view the different wounds in which this process is abused by English surgeons." What then was our chagrin and disappointment to find this important question settled by one sweeping and laconic sentence:—"I include in this number, the wound resulting from the amputation of limbs." Truly, the French exhibit to admirable perfection, in science as in politics and war, the happy talent of seasonable tergiversation! Many of our surgeons, M. Roux asserts, are utterly ignorant of the operation of Chopart for partial removal of the foot at the double articulation of the scaphoides with the astragalus, and of the cuboides with the os calcis. As "outrageous partisans of immediate re-union," we are necessarily fond of the flap operation. The plan of making a flap of the muscles of the calf of the leg, and applying it over the stump, in amputation of that member, is decried by the French surgeon as "retrograding the art." He proposes to substitute for it the double flap operation; in which the two semi-circular incisions of the soft parts unite at acute angles upon the spine of the tibia and in the middle of the calf. This method is illustrated by the narration of four cases in which it was employed by Roux, in amputations for white swelling of the ankle-joint. They all terminated favourably.

The concluding paragraph of M. Roux's work we shall now transcribe, for the amusement and edification of our readers.

"I here terminate a work which possesses a different character from what I had intended to give it. That which should have been but a simple recital of observations made upon the London hospitals, on the surgical education and practice of the English capital, has changed without my wishing, or even at first perceiving it, into a series of extended disquisitions on the principal points of surgery. Numerous practical facts are here consigned. I have never, for one moment, lost sight of the essential object of my labours, or the end which I had proposed to myself. Even when apparently deviating from it, I had always the design of rendering more perfect the parallel between English and French surgery. I have endeavoured to introduce into this parallel the most rigid impartiality. Abjuring all national prejudice and pride, I have, with equal frankness, disclosed whatever of the really fine and useful English surgery exhibits, and pointed out its defects. If it be necessary to conclude by a summary opinion, I would say, that with respect to our art,—to its habits and institutions, in whatever light contemplated, England is the land of contrasts. With features the most splendid, English surgery exhibits glaring imperfections. French surgery is most generally good."

And now for our decision on the merits of M. Roux.—Upon the whole, then, his "Narrative," although destitute of the animated and interesting tone of Mr. Cross' "Sketches," displays, we think, a more profound and philosophic character. It is evidently the production of an observant and reflecting mind, deeply versed in the principles of surgical science, and familiar with their application to practice. The task which he has imposed upon himself, is of a nature so peculiarly arduous and delicate, that few men, perhaps, of any country, and very few, most certainly, of the French, possess the qualifications requisite for its successful execution. Nothing is more easy than to profess impartiality,—nothing more difficult than to practise it, upon a subject in which all the pride and vanity of the human heart are implicated, and awakened into action. Nor can we more correctly illustrate our general notions of the performance before us, than by recording an unlucky anecdote which has just arisen on our recollection, and of which we shall leave our readers (among whom, we hope for the honour of including M. Roux himself) to make the application.—A celebrated portrait-painter once paid a visit to a neighbour in the avowed design of delineating his likeness; and with many modest protestations of his singular accuracy and talent, entered upon the work. He, however, doubtless by accident, placed himself opposite to a large mirror, which reflected the beauties of his person; and intoxicated, like Narcissus of old, by the contemplation, forgot at once his engagement and the professed object of his pencil: and the impatient neighbour, on rising from an irksome confinement, had the mortification to behold a finished painting, not of himself, but of the vain artist; and merely a coarse and distorted outline of his own phiz traced on a corner of the canvas.

The style of delineation which the "narrative" displays is embarrassed, heavy, obscure, insufferably wearisome.

The translation, lastly, is a wretched mess;—such an impenetrable combination of darkness and chaos, as completely sets at defiance all our powers of illumination and arrangement. Not a page, not a period, and scarcely a line, but presents some violation of grammar, some sin against sense or the fidelity of translation!* Like the bird of Noah in the deluge, our charity went forth in vain. No cheering speck of vegetation is to be descried amid the waste of waters; no point of *terra firma* on which it might repose; nothing to be seen but cloud and ocean: every trace of intellectual life and solidity lost in the general wreck.

---

### *Mr.* Bedingfield's *Compendium of Medical Practice, &c.*

* "These kind of ulcers, an affection not very common amongst people of the higher ranks, is one of those cases of surgery which occur but seldom in private practice, are, on the contrary, very frequent amongst the inferior classes of society." *Translation,* p. 137.—"As soon as the infiltration of urine, or urinary abscess, has rendered necessary a deep opening in the perinæum, to clear away the obstacle in the urethra, is no longer of so much importance, as when there exists only retention, but retention *complete* of the urine." *Translation,* p. 267.——From these two precious specimens, the reader, who is at all conversant with the common rules of literary composition, will be enabled to appreciate at once the extraordinary merits of the English writer. The translation of Orfila's Toxicology was bad enough; this of M. Roux's book is absolutely a disgrace to English literature. They are both, if we mistake not, productions of the same blundering hand.—Rev.

